# Book Notices

**Published:** 1895-12

**Authors:** 


### Book Notices.

TWENTIETH CENTURY PRACTICE. An International Encyclopedia of Modern Medical Science. By Leading Authorities of Europe and America. Edited by Thomas L. Steadman, M. D., New York City. In twenty volumes. Volume IV. Diseases of the Vascular System and Thyroid Gland. New York: William Wood & Co. 1895.

The number of subjects treated of in this volume are few, but they are important ones, and the very able articles here presented merit the careful perusal of the physician and student.

The first article is from the pen of Prof. James T. Whittaker, M. D., of Cincinnati, on the subject of Diseases of the Heart and Pericardium.

The exhaustive character of this article may be judged by the fact that it fills four hundred and fifty pages of this volume, and the author's well-known reputation as an able medical writer is a sufficient guarantee of its superior merit, both in subject matter contained and as a literary production. Every malformation and diseased condition of the heart and pericardium is carefully considered, and the latest and most approved treatment is suggested.

Of the four hundred and fifty pages given to the article, the diseases of the pericardium take about sixty, and the diseases of the heart itself, with its lining membrane, the balance. An interesting section is that on neuroses of the heart, to which some seventy pages are devoted. The section on valvular affections also deserves special mention.

Immediately following this article comes one on Diseases of the Blood-Vessels, by Dr. A. Ernest Sansom, of London, who is well known as an authority on these affections, This paper, of about one hundred pages, is followed by one on Diseases of the Lymphatic Vessels, by Dr. Bertrand Dawson, also of London.

The closing article, and one of special contemporaneous interest, is by Dr. George Murray, of Newcastle-on-Tyne, on Diseases of the Thyroid Gland, including Myxœdema, Cretinism, Exophthalmic Goitre, and Goitre, as well as Inflammation and Neoplasms. The editor's selection of Dr. Murray to present the subject of myxœdema is peculiarly appropriate, since it is to him that we owe the introduction of the thyroid-gland treatment which has robbed this disease and its close relative, Cretinism, of their terrors. Naturally, the author goes very fully into the history and mode of application of the thyroid treatment, taking up in their order the successive steps by which this triumph of therapeutic art has been reached. This is the first time, we understand, that the subject has ever been presented in systematic form, outside of journal articles, by the originator of the method. Some of the illustrations in this article, reproduced from photographs, showing the results of treatment, are very striking. Dr. Murray has an easy and pleasant style, and his account of this wonderful discovery is ably written.

This volume, in respect both to the importance of the subjects treated and the reputation of the authors, maintains the high standard of excellence set by those which have preceded it in the series.

THE MEDICAL NEWS VISITING LIST FOR 1896. Weekly (dated, for 30 patients); Monthly (undated, for 120 patients per month); Perpetual (undated, for 30 patients weekly per year); and perpetual (undated, for 60 patients weekly per year). The first three styles contain 32 pages of date and 160 pages of blanks. The 60 patient Perpetual consists of 256 pages of blanks. Each style is one wallet-shaped book, with pocket, pencil and rubber. Seal Grain Leather, $1.25. Philadelphia: Lea Brothers & Co., 1895.

The Medical News Visiting List for 1896 has been thoroughly revised and brought up to date in every respect. The text portion (32 pages) contains the most useful data for the physician and surgeon, including an alphabetical Table of Diseases, with the most approved Remedies, and a Table of Doses. It also contains sections on Examination of Urine, Artificial Respiration, Incompatibles, Poisons and Antidotes, Diagnostic Table of Eruptive Fevers, and the Ligation of Arteries. The classified blanks (160 pages) are arranged to hold records of all kinds of professional work, with memoranda and accounts. In the mechanical execution of the book, in quality of paper and in strength and beauty of binding nothing seems to to be left wanting. When desired, a Ready Reference Thumb-letter Index is furnished, which is peculiar to this Visiting List, and which will save many fold its small cost (25 cents) in the economy of time effected during a year. In its several styles The Medical News Visiting List adapts itself to any system of keeping professional accounts. In short, every need of the physician seems to have been anticipated in this invaluable pocket companion.

---

THE MEDICAL RECORD VISITING LIST AND PHYSICIAN'S DIARY FOR 1896. New Revised Edition. With Calendar, Tables of Doses, Tables of Equivalents, Directions for Emergencies, Antisepsis, Disinfection, Special Memoranda, Cash Accounts, etc., etc. 30 and 60 patients per week, bound in black or red morocco with flap, $1.25 and $1.50. Circular on application. William Wood & Co., publishers, New York.

This Visiting List is one of the oldest in the market, and its continued popularity shows conclusively the esteem which its convenient arrangement, good materials and attractive makeup merit. The thirty closely printed pages which precede the Visiting List proper have just been revised and brought completely up to date; aud the Tables of Equivalents, Posological Table, Tables of Formulæ for various purposes, of Poisons and their Antidotes, together with many other memoranda on subjects of daily interest to physicians, are most complete. The paper is fine, and the arrangement for recording visits and memoranda and cash accounts is excellent. The binding of the Medical Record Visiting List is of the best morocco leather, soft and flexible and extremely durable.

As usual the Medical Record Visiting List is published in two sizes, for 30 and 60 patients per week, and either dated for 1896 or without dates, so that it may be used indefiintely.

THE CREAM OF THE WRITINGS OF ALEXANDER CAMPBELL. By W. A. Morris, M. D., Austin, Texas.

This valuable book will be issued about the first of the year, bound in cloth and sheep at $2.25 and $2.50 per volume of 600 pages. While not a medical work, it has been compiled by one of the Nestors in medicine in this State, a man so well and favorably known to the profession, that a mention of his labors in this line should bring him many subscribers.

Dr. Morris has in his possession all of the numbers of the *Millennial Harbinger*, the magazine published by Alexander Campbell in the early forties, being a very rare work, and has from this source collated the best thoughts of this great thinker and put them into suitable book-form. All students of religious literature should have the work. For further information address the author at Austin.—*Texas Medical News.*

[This work rounds out in a fitting manner, and makes complete a beautiful and useful life, and merits the recognition and support or the medical profession of Texas.—ED.]

---

THE PHYSICIAN'S VISITING LIST (Lindsay & Blakiston's) for 1896. Forty-fifth year of its publication. Price, for 25 patients a week, $1; for 50 patients a week, $1.25; for 75 patients (2 vols.), $2; for 100 patients (2 vols.), $2.25. P. Blakiston, Son & Co., Publishers, 1012 Walnut St., Philadelphia.

This well known visiting list presents several improvements in the new edition for 1896.

More space has been allowed for writing the names and to the "memoranda page"; a column has been added for the "amount" of the weekly visits, and a column for the "ledger page."

To do this without increasing the bulk or price, the reading matter and memoranda pages have been rearranged and simplified.

The lists for 75 patients and 100 patients will also have special memoranda page as above, and hereafter will come in two volumes only, dated January to June, and July to December. While this makes a book better suited to the pocket, the chief advantage is that it does away with the risk of losing the accounts of a whole year should the book be mislaid.

THE ARCHIVES OF PEDIATRICS will commence its 13th year with the January number, under the business management of E. B. Treat, Publisher, of new York, long identified with medical publishing interests. The "Archives" has been for twelve years the only journal in the English language devoted exclusively to "Diseases of Children," and has always maintained a high standard of excellence.

The new management propose several important changes in its make-up; increasing the text fifteen per cent., and enlarging its scope in every way, This will give room for the fuller contributions and additional collaborators who have been secured for the various departments, all of which give promise of a more successful era than has been known even in the already brilliant career of the journal.

The editorial management will be in the hands of Floyd M. Crandall, M. D., Adjunct Professor of Pediatrics, New York Polyclynic, and Chairman of Section on Pediatrics, New York Academy of Medicine.

